# Recognition of Extracellular Bacteria by NLRs and Its Role in the Development of Adaptive Immunity

**DOI:** 10.3389/fimmu.2013.00344

**Published:** 2013-10-21

**Authors:** Jonathan Ferrand, Richard Louis Ferrero

**Affiliations:** ^1^Centre for Innate Immunity and Infectious Diseases, Monash Institute of Medical Research, Monash University, Clayton, VIC, Australia

**Keywords:** NLRs, extracellular bacteria, OMVs, adaptive immunity, innate immunity

## Abstract

Innate immune recognition of bacteria is the first requirement for mounting an effective immune response able to control infection. Over the previous decade, the general paradigm was that extracellular bacteria were only sensed by cell surface-expressed Toll-like receptors (TLRs), whereas cytoplasmic sensors, including members of the Nod-like receptor (NLR) family, were specific to pathogens capable of breaching the host cell membrane. It has become apparent, however, that intracellular innate immune molecules, such as the NLRs, play key roles in the sensing of not only intracellular, but also extracellular bacterial pathogens or their components. In this review, we will discuss the various mechanisms used by bacteria to activate NLR signaling in host cells. These mechanisms include bacterial secretion systems, pore-forming toxins, and outer membrane vesicles. We will then focus on the influence of NLR activation on the development of adaptive immune responses in different cell types.

## Introduction

A balanced relationship between humans and their microbiota is required for a variety of biological functions, including optimal protection against invasion by microbial pathogens, development of the mucosal immune system, and control of metabolic processes [reviewed in Ref. (1)]. The ability of the host immune system to distinguish between commensals and pathogens is required to avoid the development of persistent immune responses against the normal microbiota, yet maintain appropriate immune responses to pathogens. However, bacterial pathogens are able to avoid or subvert the host immune system to promote their survival and colonization. To this end, bacteria can either secrete different components into the extracellular medium or inject molecules into the host cell cytoplasm. In parallel, host cells have developed a wide range of pattern-recognition receptors (PRRs), including Toll-like receptors (TLRs) and Nod-like receptors (NLRs), to detect microorganism- and/or danger-associated molecular patterns (MAMPS and DAMPS, respectively) present in the extracellular medium or in their cytoplasm. MAMPS include virulence factors, but also essential components of both commensals and pathogens, e.g., lipopolysaccharide (LPS), peptidoglycan, or nucleic acids. Recent studies have shown that the recognition of the microbiota that takes place in the gut is necessary for the development of a normal epithelium, by controlling the balance of proliferation and differentiation, as well as maintaining a properly functioning immune system (1, 2).

Over the previous decade, the general paradigm was that extracellular bacteria were only sensed by cell surface-expressed TLRs, whereas cytoplasmic sensors, including members of the NLR family, were specific to pathogens capable of breaching the host cell membrane. Structurally, NLRs share a typical tripartite architecture with a conserved central nucleotide-binding domain, which restrains the catalytic activity of NLR family proteins. This central domain is named NACHT after the original proteins which defined the features of this domain: neuronal apoptosis inhibitory protein (NAIP), MHC class II transcription activator (CIITA), incompatibility locus protein from *Podospora anserine* (HET-E), and a telomerase-associated protein (TP1). At the C-terminal region of NLR proteins are a series of leucine-rich repeats (LRRs) that are believed to initiate NLR activation after recognition of the appropriate signal, although this mechanism is still unclear (3). The N-terminal effector domain, which specifies the function of NLRs, is less conserved. Indeed, NLRs may harbor either a pyrin domain (PYD), a caspase-activation and recruitment domain (CARD), a baculovirus inhibitor of apoptosis domain (BIR), or an as yet characterized domain (Table 1) (4). To date, 23 NLR family members have been reported, each playing different roles in pathogen recognition, homeostasis, apoptosis, or gut development [reviewed in Ref. (4)]. In the context of host-pathogen responses, NLR activation has been shown to induce the production of pro-inflammatory effectors through either nuclear translocation of Nuclear Factor-κB (NF-κB) or formation of high-molecular-weight platforms, named inflammasomes, which activate caspase-1. The main substrates of caspase-1 are cytokine pro-forms of IL-1β and IL-18, which are usually expressed in an NF-κB-dependent manner. Hence, to be expressed in a fully mature form, these cytokines require regulation at both transcriptional and post-transcriptional levels. Several distinct inflammasomes have been described in the literature, consisting of different scaffolding proteins of the NLR or the PYHIN (PYRIN and HIN-200) superfamilies [reviewed in Ref. (5)].

**Table 1:** **NLR family members and bacterial recognition**.

Effector domain	NLR family member	Bacteria	NLRs ligands	Activation mechanism	Reference
CARD	NOD1	*H. pylori*	Peptidoglycan	T4SS	Viala et al. (6)
		*H. pylori*	Peptidoglycan	OMVs	Kaparakis et al. (7), Bielig et al. (8), Chatterjee and Chaudhuri (9)
		*P. aeruginosa*			
		*N. gonorrhoeae*			
		*V. cholerae*			
		*S. typhimurium*		T3SS	Keestra et al. (10)
	NLRC4/NAIP2^a^	*S. typhimurium*	PrgJ	T3SS	Kofoed and Vance (11), Zhao et al. (12)
		*B. pseudomallei*	BsaK		
		*E. coli*	EprJ, EscI		
		*S. flexneri*	MxiI		
		*P. aeruginosa*	*Psc*I		
	NLRC4/NAIP5^a^	*S. typhimurium*	Flagellin	T3SS	Kofoed and Vance (11), Zhao et al. (12)
	NLRC4/NAIP	*C. violaceum*	CprI		Zhao et al. (12)
	NLRP12	*Y. pestis*	?	T3SS	Vladimer et al. (13)
PYR	NLRP3	*N. gonorrhoeae*	Lipooligosaccharide	OMVs, LOS	Fisseha et al. (14), Duncan et al. (15)
		*L. monocytogenes*	Listeriolysin O	PFT	Gurcel et al. (16), Mariathasan et al. (17), Harder et al. (18), Munoz-Planillo et al. (19), Dunne et al. (20), McCoy et al. (21), McCoy et al. (22), McNeela et al. (23), Kebaier et al. (24), Holzinger et al. (25)
		*S. aureus*	Hemolysins and PVL		
		*A. hydrophila*	Aerolysin		
		*A. veronii*	Aerolysin		
		*B. pertussis*	CyaA		
		*S. pneumoniae*	Pneumolysin		
		*S. pyogenes*	Streptolysin O		
		*V. vulnificus*	HlyA		
		*V. cholerae*	MARTX		
BYR	NLRP1	*B. anthracis*	Anthrax lethal toxin		Boyden and Dietrich (26)

NLR family members are classified depending on the function of the N-terminal effector domain: CARD, caspase-activation and recruitment domain; PYD, pyrin domain; BIR, baculovirus inhibitor domain.

^a^Indicates that proteins are expressed in mice only. The ligands and the activation mechanisms are detailed in the text.

Despite their intracellular localization, it has become apparent that NLRs play key roles in the sensing of not only intracellular, but also extracellular bacterial pathogens or their components. In this review, we will summarize the mechanisms used by extracellular pathogens to deliver bacterial components into host cells and how infections by these microorganisms are sensed via NLRs. Lastly, we will discuss the importance of the activation of innate immunity receptors, such as NLRs, in tailoring an appropriate adaptive immune response.

## Mechanisms Where by Bacterial Components Gain Access to the Cytoplasm

### Bacterial secretion systems

**Figure 1:**
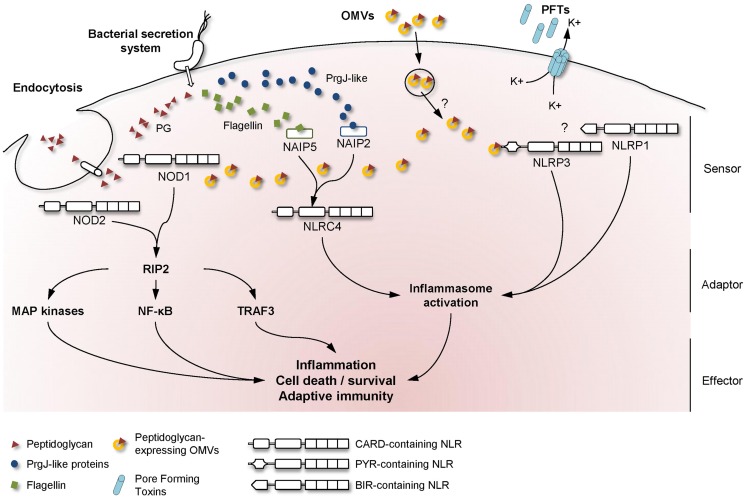
**Mechanisms used by bacteria to activate NLR signaling in host cells**. A schematic overview of the major NLR signaling pathways activated during bacterial infection, showing the mechanisms whereby extracellular MAMPs are sensed by intracellular NLRs. Upon detection of the appropriate signal, NLRs are believed to oligomerize and recruit adaptor proteins to transduce the signal to downstream effector proteins.

Bacteria that need to deliver their effectors across both bacterial and cell membranes have developed highly specialized secretion systems to reach their cytoplasmic targets. Among the six described secretion systems, the injection of bacterial components through either type-3 or type-4 secretion systems (T3SS and T4SS, respectively) has been reported to result in the activation of NLR signaling in host cells (Figure 1).

The T3SS, or “injectisome,” is a specialized molecular machine which is closely related to the bacterial flagellar apparatus. T3SSs have been identified in numerous Gram-negative bacteria, including pathogens, symbionts, and commensals, suggesting that the T3SS is not a hallmark of pathogenic microorganisms [reviewed in Ref. (27) and (28)]. The global architecture of injectisomes is conserved between bacteria and comprises a needle complex, composed of two pairs of rings that are connected by a rod, spanning the inner and outer bacterial membranes. This structure has at its end a hollow needle, a filament, or a pilus. The main function of T3SSs is to deliver effector proteins across the membranes of host cells, in which these molecules are able to activate cell signaling pathways.

Similarly, T4SSs are specialized macromolecular machines that can deliver DNA and/or proteins to host cells. In contrast to T3SSs, however, those of the type-4 family are believed to be related to bacterial conjugation systems, rather than the flagellar apparatus. T4SSs have been identified in many Gram-negative and -positive bacteria, as the complex can span both types of membrane [reviewed in Ref. (28) and (29)]. T4SSs are classified as Type A or B, depending on structure composition, but both aim to deliver bacterial effectors to host cells. The Type A T4SSs are defined by their homology with the VirB/D4 system of the plant pathogen *Agrobacterium tumefaciens*, whereas the Type B T4SSs are closer to the conjugal transfer systems of the self-transmissible IncI plasmid (29).

The first study to report a mechanism for the recognition of extracellular bacteria by intracellular receptors was described by Viala et al. who showed how virulent *Helicobacter pylori* strains are able to activate the cytosolic NLR family member, nucleotide-binding oligomerization domain-containing protein 1 (NOD1) (6). Specifically, the authors showed that *H. pylori* strains with a functional T4SS were able to deliver degradation products of Gram-negative cell wall peptidoglycan, identified as potent activators of NOD1 signaling (30), to epithelial cells (Table 1). The mechanism of how *H. pylori* peptidoglycan is delivered to cytosolic NOD1, however, is still unclear, even if it was reported that depletion of cholesterol-rich domains, or lipid rafts, interferes with peptidoglycan delivery into host cells (31). In any case, *H. pylori* T4SS-dependent induction of the NOD1 pathway was shown to result in the downstream activation of NF-κB and MAP kinases, most likely through the recruitment of the serine-threonine kinase adaptor molecule, RIP2 (32, 33). These findings are consistent with the general view that the NOD1 signaling pathway converges on the master transcriptional regulator, NF-κB, leading to pro-inflammatory cytokine production [reviewed in Ref. (34)]. One group, however, suggested that NOD1 signaling is largely independent of NF-κB and MAPK activation (35). Instead, these workers presented data showing that the dominant response mediated by NOD1 activation involves the formation of a complex known as IFN-stimulated gene factor 3 (ISGF3) and production of CXCL10 and type I IFN by epithelial cells (35). The authors demonstrated that stimulation of AGS gastric epithelial cells with either a synthetic NOD1 agonist or live *H. pylori* bacteria alone induced increased CXCL10 production. Nevertheless, the potential link between NOD1 and type I IFN in epithelial cell responses, though interesting, awaits confirmation by other researchers.

Since the work on the role of NOD1 in *H. pylori* sensing, other bacterial pathogens have been identified as also being activators of this pathway, e.g., *Pseudomonas aeruginosa* and *Campylobacter jejuni* (36, 37). These bacteria have essentially extracellular lifestyles, although some data suggest that NOD1 activation in these infection models may be due to the presence of intracellular bacteria. However, even if invasive *P. aeruginosa* and *C. jejuni* have been shown to be present in defined intracellular structures, peptidoglycan delivery, and activation of cytoplasmic sensors, such as NOD1, must still require an efficient secretion system (38, 39). Furthermore, a recent study has shown that *Salmonella typhimurium* activation of NOD1 and NOD2, a related molecule that senses all forms of bacterial peptidoglycan, may be invasion-independent but requires an intact T3SS for the injection into the cytoplasm of the bacterial protein, SipA (10). This work, identifying a novel agonist of NOD1- and NOD2-signaling, suggests that these NLR family members may be activated through a distinct pathway from that induced by bacterial peptidoglycan. The same group suggested recently that NOD1 could sense other patterns of pathogenesis, such as modification of the actin cytoskeleton. According to this intriguing new model, a T3SS-secreted protein of *S. typhimurium*, SopE, activates the small Rho GTPases, which then triggers the NOD1 signaling pathway (40).

Another major NLR family member that is activated by extracellular pathogens through the action of bacterial secretion systems is the CARD-containing protein, NLRC4 (previously known as Ice Protease-Activating Factor, IPAF) (Table 1). The early paradigm for NLRC4 activation was that this NLR senses bacterial flagellin within the cytoplasmic compartment of cells. Specifically, it was shown that T3SS-dependent translocation of *S. typhimurium* and *P. aeruginosa* flagellin into the host cell triggered NLRC4 activation in macrophages (41–44). Interestingly, subsequent reports contradicted this hypothesis as both *Shigella flexneri*, an aflagellated bacterium, and *P. aeruginosa* were also able to activate NLRC4 inflammasome in a flagellin-independent, but T3SS-dependent mechanism, suggesting the existence of one or several other ligands (45, 46). These findings have since been confirmed in various bacterial species and the molecules responsible for NLRC4 activation have now been identified. Thus, it was shown that the basal body rod components of T3SSs (rod protein) are detected during infection with either *S. typhimurium* (PrgJ), *Burkholderia pseudomallei* (BsaK), *Escherichia coli* (EprJ and EscI), *S. flexneri* (MxiI), or *P. aeruginosa* (*Psc*I) (47). Interestingly, all these proteins appear to share a sequence motif present in the carboxy terminal region that is conserved in the flagellar protein, FliC, and detected by NLRC4 (47). The mechanism of how NLRC4 is able to sense and respond to two distinct bacterial products, flagellin or PrgJ-like proteins, has thus been deciphered. Concomitant animal infection studies from two different groups showed that the specificity of the NLRC4 inflammasome is determined by different NAIP paralogs (11, 12). In these new models, both flagellin and rod protein are injected through the T3SS and are specifically recognized by NAIP5/6 or NAIP2 respectively, confirming previous studies showing a physical association between NLRC4 and NAIP5 (Figure 1) (11, 12, 48). Interestingly, humans possess only one *Naip* gene compared with the six *Naip* genomic loci in mice, and the specificity of human NAIP appears to be different to the murine NAIPs as it is unresponsive to intracellular delivery of flagellin or PrgJ-like rod proteins (12). Although it has been shown that a PrgJ homolog, from the bacterium *Chromobacterium violaceum*, is specifically recognized by human NAIP (12), further studies are required to determine the importance of human NAIP during infection with common bacterial pathogens.

T4SS and flagellin have also both been suggested to play roles in NLRC4 activation during *Legionella* replication within macrophages, since the presence of all three elements (i.e., flagellin, a functional T4SS and NLRC4) is required to activate caspase-1 in these cells. Consistent with these findings, *in vivo* studies confirmed that clearance of *Legionella pneumophila* required the presence of both flagellin and NLRC4 (49).

Finally, bacterial secretion systems have recently been implicated in the activation of the NLR PYD-containing protein 12 (NLRP12) by the pathogen, *Yersinia pestis* (Table 1) (13). The nature of the NLRP12 ligand is still unknown, however, by using a *Y. pestis* strain which lacks the virulence plasmid necessary for the formation of a T3SS, the authors were able to show that this secretion system was required for inflammasome activation and IL-1β release in bone marrow-derived macrophages (BMDMs) (13). The precise mechanism by which the *Y. pestis* T3SS mediates NLRP12 inflammasome activation remains to be elucidated.

### Outer membrane vesicles

In addition to bacterial secretion systems, in which individual proteins or macromolecules are secreted, bacteria have developed other mechanisms to transfer a wide variety of components within the host cells. The release of outer membrane vesicles (OMVs) is one such strategy developed by Gram-negative bacteria to secrete toxins, enzymes, DNA, adhesins, or other periplasmic constituents into the extracellular medium [reviewed in Ref. (50)]. It is also noteworthy that the commensal bacterium, *Bacteroides fragilis*, releases a capsular polysaccharide in its OMVs that has immunomodulatory effects and can prevent inflammation in an experimental colitis model (51). OMVs are released by virtually all Gram-negative bacteria, whereas the level of expression differs between bacterial species (50). More recent reports also suggest that Gram-positive bacteria can secrete membrane vesicles, however, these are less well studied than those of Gram-negative organisms (52, 53). OMVs can be released under different conditions *in vitro* and *in vivo* from free-living cells, biofilms, or by internalized bacteria (54–56).

The primary roles of OMVs are believed to be the delivery of toxins or bacterial components into host cells and the evasion of host immune responses (57). On the other hand, several studies have revealed that OMVs are able to induce inflammatory responses that may protect the host from infection. Indeed, OMVs contain different MAMPs, including LPS, flagellin, or DNA that could be recognized by TLR and NLR family members (51, 58, 59). Traditionally, many studies in the literature have focused on OMV-associated LPS, however, it is now becoming apparent that peptidoglycan represents a major promoter of the inflammatory responses induced by OMVs. An initial clue to the potential role of OMV-associated peptidoglycan in innate immunity arose from the observation that *S. flexneri* culture supernatants, when microinjected into epithelial cells, induced the activation of the NF-κB signaling pathway (30, 60). Indeed, it was shown that the OMVs normally present in such supernatants are able to enter non-phagocytic cells and deliver peptidoglycan directly to cytoplasmic NOD1, resulting in the up-regulation of NF-κB and IL-8 responses in cells (Figure 1) (7). This OMV-dependent mechanism of NOD1 activation was demonstrated for three extracellular pathogens: *H. pylori*, *P. aeruginosa*, and *Neisseria gonorrhoeae* (Table 1) (7). Moreover, it was shown that Nod1-deficient mice intragastrically fed with *H. pylori* OMVs failed to mount local *Cxcl2* and systemic antibody responses, when compared with their wild-type littermates (7).

Subsequent studies reported that *Vibrio cholerae* strains, which also produce large amounts of OMVs, can promote immune responses in the host via a NOD1- and NOD2-dependent mechanism (8, 9). OMVs isolated from *Moraxella catarrhalis* have also been shown to induce IL-8 production through TLR2-initiated NF-κB activation, but a role for NOD1 could not been excluded, as cellular responses to whole bacteria involved both TLR2 and NOD1 (61, 62). Pro-inflammatory responses to OMVs were also observed for several other bacteria [reviewed in Ref. (50)], however, the role of peptidoglycan in these responses was not assessed.

Besides peptidoglycan, OMVs contain other molecules able to activate the innate immune system. For example, *N. gonorrhoeae* OMVs contain lipooligosaccharide (LOS) which has been shown to activate NLRP3-induced IL-1β secretion and pyronecrosis in monocytes and macrophages (Figure 1) (14, 15). This LOS-mediated activation of the NLRP3 inflammasome is believed to be triggered by the release of cathepsin B (15).

Mechanistic data on OMV uptake are scarce, but recent findings suggest that disruption of lipid rafts by treatment with Fumonisin B_1_, an inhibitor of sphingomyelin incorporation into lipid rafts, or methyl-β-cyclodextrin, a cholesterol-depleting agent, abrogates both internalization and NOD1-dependent immunostimulatory capacity of OMVs (7). The mechanism and intracellular compartment(s) involved in NOD1 sensing of OMV-associated peptidoglycan, however, have yet to be determined.

The findings concerning the role of peptidoglycan delivery by OMVs provide a new mechanism to understand how extracellular bacteria, which are unable to invade cells or to inject components through a secretion system, may be able to initiate innate immune signaling in non-phagocytic cells, such as epithelial cells.

### Pore-forming toxins

In addition to OMVs, bacteria may secrete toxins to alter host cell integrity distant to the original point of invasion. Among these proteins are the pore-forming toxins (PFTs), which are produced by a wide range of pathogens. PFTs are secreted in a soluble form and are subsequently multimerized into a transmembrane channel that perforates the plasma membrane of host cells. Pore formation may be an entry door for bacterial molecules to penetrate into host cells or lead to cellular ion imbalance (63). Efforts during the last decade have been focused on determining how cells are able to mount a response against pore formation, thereby contrasting with the existing paradigm, which suggested that cells possessed no defenses against these toxins and that the only outcome was cell death (63, 64). Numerous studies, indeed, found that stimulation of immune cells with different PFTs activates pro-inflammatory signaling or vacuolation in response to treatment [(65); and reviewed in Ref. (64)]. It is also noteworthy that the concentrations of PFTs during *in vivo* infection could be sublytic, thus allowing cells to mount an antibacterial response (66), capable of controlling the infection.

Recent intensive studies on PFTs and cellular responses have revealed a major role for NLRP3 in the sensing of pore formation. Mariathasan et al. demonstrated a role for listeriolysin O from *Listeria monocytogenes*, as well as for an unknown *Staphylococcus aureus* toxin, in inflammasome activation and IL-1β production (Table 1). Furthermore, this study speculated that the observed effects were dependent on intracellular potassium levels (Figure 1) (17). Concerning *S. aureus*, recent studies showed that caspase-1 activation requires the presence of all three of its α-, β-, and γ-hemolysins and the release of bacterial lipoproteins (19, 24). In addition, a small percentage of *S. aureus* isolates also produce another toxin, named Panton–Valentine leukocidin (PVL), which is able to trigger NLRP3 inflammasome activation (25). These results were confirmed using aerolysin from *Aeromonas hydrophila* which was shown to mediate the efflux of intracellular potassium ions and activation of caspase-1 through the assembly of NLRC4 and NLRP3 inflammasomes (16). Subsequent studies suggested that aerolysin from either *A. veronii* or *A. hydrophila* activates only the NLRP3 inflammasome, through potassium efflux, whereas NLRC4 activation was T3SS-dependent but potassium-independent (21, 22). During the last decade, a large number of bacterial PFTs have been shown to be able to activate NLRP3 via the same molecular mechanism of potassium efflux. These PFTs include the adenylate cyclase toxin (CyaA) from *Bordetella pertussis* (20), pneumolysin from *Streptococcus pneumoniae* (23), HlyA hemolysin and MARTX from *Vibrio vulnificus* and *V. cholerae* (23), streptolysin O from *Streptococcus pyogenes* (18). Even if the molecular mechanism is unclear, NLRP3 inflammasome assembly occurs spontaneously at low potassium concentrations and is prevented at higher concentrations, thus confirming its role in the detection of DAMPs (67). In a recent study, potassium efflux was shown to be the minimal membrane permeabilization event triggering NLRP3 inflammasome activation by PFTs and particulate matter (68).

In contrast to our understanding of NLRP3 biology, far less is known about activation of the NLRP1 inflammasome and its activation by PFTs. The human *Nlrp1* gene has three murine paralogs, which encode proteins lacking the N-terminal PYD sequence found in human NLRP1 (69). Sensitivity of mice to the effects of anthrax lethal toxin from *Bacillus anthracis* has been correlated with a polymorphism in the *Nalp1b* gene, encoding Nlrp1 (26). Due to the absence of a PYD domain in the mouse sequence of Nlrp1, it is not clear whether caspase-1 recruitment requires ASC or dimerization with another NLRP. However, recent data suggest that upon stimulation with anthrax lethal toxin, NLRP1 undergoes autoproteolysis to form an inflammasome (70). Interestingly, inflammasome formation and caspase-1 recruitment are inhibited by high levels of potassium (71). Hsu et al. demonstrated a role for NOD2 in lethal toxin-induced IL-1β production and suggested the formation of a complex between NLRP1 and NOD2 (72). This association has since been confirmed by other authors (73). Interestingly, NOD2-recognition of *S. aureus* is facilitated by the presence of its hemolysin, probably by promoting cytoplasmic access of NOD2 ligand (74).

Synergistic responses from the activation of multiple NLR pathways have been observed following co-stimulation with two different pathogens. Indeed, it was shown that *Haemophilus influenzae* peptidoglycan enters epithelial cells more efficiently in the presence of *S. pneumoniae* pneumolysin, suggesting the ability of intracellular NLRs to sense extracellular bacteria that do not encode secretion systems or express OMVs or PFTs (75). One hypothesis from the authors is that host organisms have evolved to detect a combination of pathogens in order to mount optimal responses in ways that are different to the responses induced by a single infection. It is now recognized that co-infection plays a previously unappreciated yet important role in the development of mucosal immunity and disease progression (76).

In conclusion, increasing numbers of studies suggest that osmotic changes induced by pore formation may be sensed by intracellular NLRs as an early warning system (68). Furthermore, these signals can cooperate with other signaling pathways, thus leading to the generation of antibacterial responses before the concentration of PFTs reaches a lytic concentration.

### Endocytosis

Asides from the active processes described above, intracellular NLRs may be activated by passive mechanisms. One such mechanism involves cellular entry by the peptidoglycan fragments that are released during bacterial growth or are degraded by host enzymes. Bacteria express peptidoglycan-degrading enzymes, necessary for maintaining functional growth, division, and development [reviewed in Ref. (77, 78) and (79)]. In addition to their role in shaping bacterial membranes, these enzymes are responsible for the release of free peptidoglycan fragments in the extracellular compartment. It is thus possible that these fragments interact with surrounding organisms, mediating pathogenic effects on host cells, as mutations in peptidoglycan-recycling proteins result in decreased pathogenesis. Conversely, released peptidoglycan fragments can also play a role in symbiotic relationships, such as is the case with *Vibrio fischeri*, which induces developmental changes in its squid host [reviewed in Ref. (77)]. Moreover, peptidoglycan can have effects on host cells distant to the point of its release. For example, peptidoglycan released in the gut was shown to circulate and play a major role in the priming of neutrophils in the bone marrow (80). Interestingly, a study by Hasegawa et al. characterizing NOD1- and NOD2-stimulatory activities in different bacterial preparations, showed that the highest levels of NOD1-stimulatory activity were found predominantly in culture supernatants, whereas NOD2 activity was associated with extracts from whole bacterial cells (81). This study further underscores the likely important role of released peptidoglycan during infection with extracellular bacteria.

On the other hand, host cells have also developed some mechanisms to degrade bacterial peptidoglycan in order to kill invading pathogens and provide ligands to host receptors [reviewed in Ref. (82)]. Enzymes such as lysozyme or peptidoglycan recognition protein family members generate fragments small enough to be sensed by NOD1 and NOD2 (82, 83).

In the context of extracellular pathogens, we can then wonder how these peptidoglycan fragments are processed to be presented to intracellular innate immune sensors. It is now established that different internalization mechanisms can be used by the host cell and these are probably cell-type dependent. In the case of epithelial cells, it has been reported that NOD1 and NOD2 ligands are likely to be internalized by clathrin-mediated endocytosis (84, 85). In immune cells, it is more likely that internalization occurs through phagocytosis. Interestingly, it has been observed that Nod1- or Nod2-deficient mice have decreased phagocytic abilities (86, 87).

Once the fragments have been internalized in vesicles by clathrin-mediated endocytosis or phagocytosis, they have to be delivered across membranes and be presented to the cytosolic molecules, NOD1 and NOD2. This mechanism implies the presence of specific transporters, with the transporter family SLC15 having been proposed to play a role. This transporter family comprises membrane proteins controlling the cellular uptake of di/tripeptides and peptide-like drugs [reviewed in Ref. (88)]. Roles for SLC15A4 (PHT1) and SLC15A2 (PepT2) have indeed been identified for the delivery of NOD1-ligands (Figure 1) (84, 89, 90). Another member of this family, SLC15A1 (PepT1), is believed to play a role in NOD2 ligand transport (85, 91). Nevertheless, the specificity of each of these transporters is still unclear, as both the minimal motif recognized by NOD1, iE-DAP (γ-d-Glu-mDAP), and the NOD2 agonist, MDP (MurNAc-l-Ala-d-isoGln, also known as muramyl dipeptide) may be delivered through SLC15A2 (89, 92). Interestingly, there are higher expression levels of SLC15A1, SLC15A2, and SLC15A4 in the small intestine, with lower bacterial loads of 10^3^ organisms per gram, than in the colon, where microbial densities reach 10^12^ organisms per gram. This may suggest a role for such transporter proteins in MAMP uptake [reviewed in Ref. (93), and (1)].

## Control of Adaptive Immunity

Activation of NLRs has been shown to prime T and B cells, suggesting the contribution of these innate immune molecules in the development of adaptive immune responses. Interestingly, many of the known NLRs activators (e.g., MDP, flagellin, alum) play roles as adjuvants during vaccination, suggesting a role for these molecules in tailoring the adaptive immune response [reviewed in Ref. (94)].

The most striking example is the role played by NOD2 in the mediation of the adjuvant effect of complete Freund’s adjuvant, first described in 1937 (95). The adjuvant activity of this compound, which is composed of paraffin oil and *Mycobacterium tuberculosis* extract, is believed to be attributed to its ability to prolong antigen release, to promote the recruitment of immune cells and antigen presentation by inducing expression of cytokines and chemokines [reviewed in Ref. (96)]. The minimal component of complete Freund’s adjuvant was subsequently identified to be MDP, as this molecule provided the same level of adjuvant activity as whole killed *M. tuberculosis* (97). The mechanism responsible for the activity of Freund’s adjuvant was determined less than 10 years ago by Kobayashi et al. who showed that NOD2 was required for the development of protective immunity mediated by the adjuvant effects of MDP (98). During immunization assays in Nod2^−/−^ animals, a severe deficiency was observed in the production of antigen-specific immunoglobulins, specifically in those of the IgG1 subclass, suggesting that NOD2 is able to activate the adaptive immune system and promote the production of antibodies to T cell-dependent antigens (98). These results were confirmed subsequently, when it was shown that MDP injection in mice triggered Th2 polarized responses in a NOD2-dependent manner (99). Specifically, it was shown that Nod2-deficient mice displayed impaired chemokine and Th2 responses with low numbers of splenic IL-4- and IL-5-producing T cells, as well as the loss of antigen-specific T and B cell responses (99). Interestingly, NOD2 can also cooperate with TLRs to generate Th1-polarized responses to co-stimulation with MDP and TLR2 or TLR4 ligands, suggesting the importance of complementary effects between TLRs and NLRs in the balance of immune effector responses (99). In addition to the recognition of MDP by NOD2, dual recognition of a mycobacterial glycolipid, also known as cord factor, and peptidoglycan is essential for Th17-differentiation in an inflammasome-dependent manner (100). It was shown that recognition of both the mycobacterial cord factor by the CARD9-dependent C-type lectin receptor mincle and peptidoglycan, via a NOD1- and NOD2-independent mechanism, induces inflammasome activation and IL-1β secretion and thus drives skewed Th17 responses.

NOD1 stimulation has also been shown to be sufficient to drive antigen-specific immunity with a predominant Th2 polarization profile and to play a role in the onset of Th1, Th2, and Th17 immune pathways in conjunction with TLR stimulation (101). Thus, depending on the presence of different MAMPS and the co-stimulation of TLRs, NOD receptors can initiate different arms of the adaptive response. Although data are still scarce concerning the role of NOD1 and NOD2 in the onset of adaptive immunity during microbial infection, *H. pylori*-infected Nod1-deficient mice exhibited reduced Th1 immune responses compared with their WT littermates (101). Similar results were obtained in *M. tuberculosis* or *S. pneumoniae*-infected Nod2-deficient mice, with lower titers of pathogen-specific serum IgG and diminished antigen-specific T cell responses (102, 103). Consistent with these data, *Citrobacter* and *Salmonella* infections triggered Th17 responses that were dependent on NOD1 and NOD2 (104).

Injection of bone marrow reconstituted mice with NOD1 or NOD2 agonists and ovalbumin allowed the group of Philpott and collaborators to determine the importance of stromal factors, versus hematopoietic cells, in the initiation of Th2 immune responses (101, 105). In addition, that group showed that the capacity of NOD1 ligand to cooperate with TLR agonists was completely abolished in Nod1-deficient bone marrow-derived dendritic cells (BMDCs) (101). Similarly, Nod2-deficiency in BMDCs abolished pro-inflammatory cytokine production upon stimulation with MDP alone, whereas synergy between MDP and TLR ligands was lost in Nod2-deficient BMDMs (98). Additionally, full responses required sensing within the hematopoietic compartment, with a major role for dendritic cells, consistent with the well-established role of dendritic cells in the onset of adaptive immunity (105, 106). Furthermore, co-stimulation with NOD1 or NOD2 agonists in combination with TLR agonist induced a synergistic production of Th1-associated cytokines IFN-γ and IL-12 (107).

In contrast to the now well-defined role of NOD proteins in tailoring adaptive immune responses, the role of the NOD1/2 adaptor protein, RIP2, is less well-defined. Several early works in *in vivo* or *in vitro* RIP2-deficient models demonstrated impairment in the development of anti-infectious responses, NF-κB signaling, or T cell proliferation and differentiation (108–110). On the other hand, more recent papers, all of them using the same mouse model but different to those used previously, claimed an absence of effect of RIP2 in T cell proliferation and T helper differentiation (111, 112). A comparison of the different RIP2-knockout mouse lines may help to resolve these differences.

Recent studies have shown that non-hematopoietic cells can also be of importance during the development of adaptive immune responses. Indeed, Watanabe et al. proposed that activation of NOD1 and NOD2 in gastrointestinal epithelial cell lines induces production of cytokines associated with a Th1 response (35). They also proposed that NOD1 signaling, through ISGF3 activation and type I IFN responses, may lead to Th1 differentiation and Th1-dependent inflammation (35).

Concerning the other NLR family members, further studies will be needed to help to understand their roles in adaptive immunity. It has been shown using the Listeria infection mouse model that strains that activated the inflammasome generated significantly less protective immunity, a phenotype that correlated with decreased induction of antigen-specific T cells (113). It is noteworthy that IL-1 family cytokines, including IL-1β and IL-18, have adjuvant properties, as they can induce antigen-specific immune responses against infection (114). For example, CyaA, a pore-forming toxin from *B. pertussis*, activates the NLRP3 inflammasome and induces IL-1β expression, thereby playing a critical role in promoting antigen-specific Th17 cells and in generating protective immunity against *B. pertussis* infection (20). Interestingly, a recent report suggested that IL-1β production in trophoblasts after *Chlamydia trachomatis* infection may also be mediated by NOD1, but the signaling pathway involved remains unclear (115). In addition, IL-1β may be secreted after non-canonical inflammasome activation, where an intracellular lipid A moiety of LPS has been showed to play major roles in the induction of TLR4-independent inflammatory responses (116). Although the receptor has as yet to be characterized, these results suggest a new mechanism of intracellular sensing in the mounting of innate immune responses against microbial infection.

## Concluding Remarks

As discussed above, various NLR family members have evolved to detect infection and mount effective immune responses mediated by both innate and adaptive arms of the immune system. Asides from these NLR family members, it is possible that other family members could play roles during infection with extracellular bacteria. Indeed, NLRP6-deficiency in mice was shown to result in increased inflammation, to alter the colonic microbial ecology and was associated with susceptibility to colorectal tumorigenesis (117, 118). More recently, NLRP6 has been shown to inhibit NF-κB translocation and MAPK activation, with NLRP6 activation leading to increased susceptibility to both intracellular and extracellular bacteria (119). Thus, a second subclass of NLR family members, such as NLRP6 or NLRC5, may act as molecular switches to dampen host responses induced by extracellular bacteria. For example, NLRC5 has been suggested to interact with NF-κB regulators, IKKα and IKKβ, and to block their phosphorylation so as to modulate inflammatory signaling to bacterial pathogens (120). Discordant results were, however, obtained in different studies (121–123), suggesting that further investigations are required to fully elucidate the role(s) of NLRC5 in host responses to microbial pathogens. Besides the NLR family members described in this review, other intracellular molecules, such as certain TLRs (i.e., TLR3, TLR9) or Absent In Melanoma 2 (AIM2), are able to detect nucleic acids from extracellular bacteria, allowing a wide range of MAMPs to be sensed.

The different examples of infection sensing described above highlight the existence of dual systems of recognition for MAMPs from extracellular bacteria. First, conserved molecular patterns in bacteria may be recognized by extracellular receptors. For instance, sensing of bacterial lipoproteins by TLR2, LPS by TLR4 or flagellin by TLR5 have been relatively well described. Activation of these extracellular receptors leads to a transcriptional inflammatory response with production of type I IFNs or pro-inflammatory cytokines, such as TNF-α or IL-12. However, particular cytokines, including IL-1β or IL-18, require an additional post-transcriptional step to be fully functional. A growing amount of evidence suggests that the signaling involved in this post-transcriptional response is due to activation of inflammasome complexes, after sensing of microbes or danger signals by intracellular molecules, including the NLRs. However, some intracellular sensors of extracellular bacteria, such as NOD1 and NOD2, do not induce inflammasome formation and are generally thought to activate NF-κB signaling instead. Nevertheless, we can reasonably hypothesize that host cells are able to distinguish between the signals originating from extracellular and intracellular pathogens, through the intensity, kinetics, or cell-specific nature of the signal.

This dual recognition of the pathogen itself, or of the consequences of the infection, may be of importance to finely tune inflammatory responses in line with the threat. One hypothesis is that non-pathogenic bacteria may be recognized by extracellular receptors only, whereas pathogenic extracellular or invasive bacteria will be sensed by both families of receptors, leading to more intense responses, suggesting that synergy between TLRs and NLRs may be required for optimal responses. As evoked in this review, it was found that NLR family members may synergize with TLR-dependent cytokine expression (124). An interesting example of this possible dual recognition would be the gut, where there is exposure to more than 500 species of commensal microorganisms. It has been shown that TLR agonists induced tolerance to subsequent stimulation with the same agonist (125). This process could thus play a role in the induction of tolerance to commensal bacteria, whereas pathogenic microorganisms could then be sensed by NLR family members.

It is possible that, depending on the cell type, host cells may distinguish between the signal originating from TLRs and NLRs. Gut immunology provides a good example of how this might work. Indeed, the intestinal epithelium is composed of different layers allowing discrimination between commensal and pathogenic bacteria. The outermost of these layers, comprising the mucus, is a barrier surrounding intestinal epithelial cells. Some areas of the intestinal epithelium, such as the Peyer’s patches, are devoid of mucus and serve as inductive sites for the mucosal immune system. In addition, dendritic cells can extend pseudopodes through the mucus and reach the lumen [reviewed in Ref. (126)]. This multi-layer system could allow the host to distinguish between a commensal, which should not progress through the mucosa, and a pathogen, which could disseminate beyond this layer and/or present bacterial components to the epithelium (127). Hence, the ability of the host to distinguish between commensals and pathogens and to mount efficient immune responses could be dependent on how and where the MAMPs are sensed (128).

As discussed in this review, activation of NLR-dependent signaling pathways by extracellular bacteria induces the direct production of pro-inflammatory molecules and also tailors and drives adaptive immunity, suggesting that NLR family members are multifaceted proteins. A comprehensive understanding of the functions of NLRs will help decipher their roles in shaping both innate and adaptive immunity during infection with extracellular pathogens.

## Conflict of Interest Statement

The authors declare that the research was conducted in the absence of any commercial or financial relationships that could be construed as a potential conflict of interest.
